# CD34^+^ and CD34^−^ MM cells show different immune-checkpoint molecule expression profiles: high expression of CD112 and CD137 ligand on CD34^+^ MM cells

**DOI:** 10.1007/s12185-024-03867-0

**Published:** 2024-11-12

**Authors:** Ayano Fukui-Morimoto, Kentaro Serizawa, Ko Fujimoto, Aki Hanamoto, Yoshio Iwata, Hiroaki Kakutani, Takahiro Kumode, Chikara Hirase, Yasuyoshi Morita, Yoichi Tatsumi, Hitoshi Hanamoto, Hirokazu Tanaka, Itaru Matsumura

**Affiliations:** 1 Department of Hematology and Rheumatology, Kindai University Faculty of Medicine, 377-2, Ohno-Higashi, Osaka-Sayama, Osaka 5898511 Japan; 2 Department of Hematology, Kindai University Nara Hospital, Ikoma, Nara Japan

**Keywords:** Multiple myeloma, CD34, Myeloma-initiating cells, Immune-checkpoint inhibitors, TIGIT, CD137

## Abstract

Despite the introduction of new drugs, multiple myeloma (MM) still remains incurable. We previously reported that CD34^+^ MM cells, which are clonogenic and self-renewing, are therapy-resistant and persist as a major component of minimal residual disease, expanding during relapse. To investigate the effects of immunotherapies such as immune-checkpoint inhibitors, CAR-T therapy, and bispecific antibodies on CD34^+^ MM cells, we analyzed immune profiles of both MM cells and T cells from MM patients using microarrays and flow cytometry. Ingenuity pathway analysis revealed 14 out of 289 canonical pathways were more active in CD34^+^ MM cells compared to CD34^−^ cells, many of which were involved in inflammation and immune responses. Notably, PD-1 signaling-related genes were highly expressed in CD34^+^ MM cells. Among 10 immune-checkpoint molecules, CD34^+^ cells more frequently expressed CD112, CD137L, CD270, CD275, and GAL9 than CD34^−^ cells in both newly diagnosed and relapsed/resistant patients. In addition, CD4^+^ and CD8^+^ T cells more frequently expressed TIGIT and CD137, suggesting that CD112/TIGIT and CD137L/CD137 interactions may suppress T-cell activity against CD34^+^ MM cells. Furthermore, our finding of higher FcRH5 expression on CD34^+^ MM cells is encouraging for future research into the efficacy of FcRH5-targeted therapy in MM.

**Supplementary Information:**

The online version contains supplementary material available at 10.1007/s12185-024-03867-0.

## Introduction

Multiple myeloma (MM) is a hematologic malignancy characterized by the proliferation of clonal plasma cells in bone marrow (BM). Early genetic mutations in post-germinal center B/plasma cells are believed to cause myeloma development. Additional chromosomal aberrations and mutations further drive expansion of MM cell population [1, 2]. In addition, the growth and survival of MM cells are positively or negatively regulated by tumor microenvironment (TME), which includes the extra cellular matrix and diverse cell types such as stromal cells, osteoclasts, and immune regulatory cells (T, B, NK cells, regulatory T cells [Tregs], myeloid-derived suppressor cells [MDSC], macrophages, and etc.) [3–7].

Recent advances in MM treatment, particularly the development of novel drugs such as proteasome inhibitors (PIs; bortezomib, carfilzomib, ixazomib), immunomodulatory drugs (IMiDs; thalidomide, lenalidomide, pomalidomide), and monoclonal antibodies (mAbs; daratumumab and isatuximab targeting CD38, and elotuzumab targeting SLAMF7), have greatly improved clinical outcomes. These drugs are highly effective and can achieve deep responses (such as complete remission [CR], immunophenotyptic CR, and molecular CR) in many of MM patients. However, even in these favorable states, therapy-resistant MM cells still remain in BM as minimal residual disease (MRD), leading to relapse. After repeated cycles of remission and relapse, most of the patients consequently succumb to the disease. So, eliminating residual MM cells is critical to improve prognosis of MM [8, 9].

The advent of immune-checkpoint inhibitors (ICIs) has revolutionized cancer treatment, garnering considerable interest in their application for hematologic malignancies [10–12]. Hodgkin’s lymphoma and primary mediastinal B-cell lymphoma, in particular, has emerged as the most suitable target diseases of ICIs, showing remarkable response rates and clinical outcomes [13–16]. Although ICIs have demonstrated efficacy in some clinical trials for MM, either as monotherapy or in combination with other drugs, it has not been approved for MM due to concerns about their limited efficacy and toxicity [17]. Nevertheless, a growing body of evidence suggests that the immune response plays a crucial role in clinical outcomes in MM patients [18–20]. If appropriately tailored based on disease subtypes, patient characteristics, and/or immune profile of MM cells, ICIs could undoubtedly provide substantial clinical benefits for MM patients.

Our previously research found that CD34^+^ MM cells as being enriched in the side population (SP) of MM cells (47.8% in SP MM cells *vs*. 2.11% in bulk MM cells) [21]. Unlike CD34^−^ MM cells, CD34^+^ MM cells exhibited clonogenic activity and long-term self-renewal activity in a xenotransplantation model. While only 2.20% of MM cells were CD34^+^ in newly diagnosed MM (NDMM), this fraction increased to 42.6% in MRD samples and 17.7% in refractory/relapsed MM (RRMM). In addition, cell cycle analysis revealed that 24.7% of CD34^+^ MM cells from NDMM were in the therapy-resistant G0 phase, this proportion increased to 54.9% in MRD samples and decreased to 14.5% in RRMM samples, reflecting their expansion [21]. These results suggest that CD34^+^ MM cells persist as MRD in a quiescent state following effective treatment, or expand as therapy-resistant cells in RRMM under conventional therapies such as PIs and IMiDs [22]. So, new therapeutic strategies targeting CD34^+^ MM cells are necessary to further improve clinical outcomes and potentially cure MM.

In addition to PIs, IMiDs, and antibody (Ab)-based drugs, novel immune-based therapies, including ICIs, chimeric antigen receptor T-cell (CAR-T) therapies [23, 24], and bispecific Abs such as bispecific T-cell engagers, BiTEs) [25–27] have shown efficacy in a subset of RRMM patients. To enhance the effectiveness of these immune therapies, in the present study, we sought to elucidate the immune profile of CD34^+^ MM cells in the present study.

## Methods

### Patient samples

A total of 34 MM patients (19 NDMM, 15 RRMM) were included in this study. Patients’ characteristics are shown in Table 1. Diagnosis, remission, relapse, and resistance of these cases were defined by the International Myeloma Working Group criteria. Mononuclear cells were isolated from BM samples from MM patients. Normal healthy BM samples were obtained from patients with the similar median age to MM patients, who did not have hematologic disease as a result of the screening. Mononuclear cells were isolated with Ficoll-Paque PLUS (GE Healthcare Bio-Science AB, Uppsala, Sweden) and stored at Kindai University Faculty of Medicine until use. All samples were collected after obtaining the written informed consent from the patients. This study was approved by the ethics committee of our institute (Authorization Number: 24-017, -018) and conducted according to the Declaration of Helsinki.

**Table 1:** Characteristics of MM samples

	NDMM (*n* = 23)	RRMM (*n* = 20)
Median Age (range)	72 (48–91)	75 (61–84)
Sex, No. Male/Female	9/14	6/14
Myeloma type		
IgG-κ	9	11
IgG-λ	5	1
IgA-κ	3	3
IgA-λ	4	3
BJP-κ	1	1
BJP-λ	1	1
ISS		
I	2	2
II	4	7
III	17	11
Median % MM cells in BM (range)	33.5 (10.3–89.6)	6.7 (1.0–66.4)
G-banding		
Normal	19	15
t(2;4)	1	0
t(7;15)	1	0
t(11;18)	0	1
Complex	2	2
NA	0	2
High risk CAs		
Negative	10	1
17p del	1	2
1q gain	2	0
t(11;14)	0	1
NA	10	16
Treatment		
None	23	0
PI treated	0	4
IMiD treated	0	7
PI-, IMiD treated	0	9
anti-CD38 Ab treated	0	0
ASCT	0	3
Median interval from the last therapy to sample collection, month (range)	NA	1 (1–54)

*MM* Multiple myeloma, *BM* Bone marrow, NA Not anayzed, *CAs* chromosomal abnormalities, *PI* Proteosome inhibitor, *IMiD* Immunomodulatory drug, *Ab* Antibody, *ASCT* Autologous stem cell transplantation

### Flow cytometric analysis and cell sorting

After incubation with an FC blocking reagent, MM cells were stained with the appropriate Abs as listed in the Supplemental Methods for 30 min at 4 ℃. Cytoplasmic immunoglobulin was detected with a BD Cytofix/Cytoperm Fixation/Permeabilization Solution Kit (BD Biosciences, San Jose, USA). All flow cytometricy analyses and cell sorting were performed on a BD FACS AriaIIcell sorter instrument (BD Biosciences).

### Microarray analysis and gene set enrichment analysis (GSEA)

RNA was extracted with PureLink® RNA Mini Kit (Thermo Fisher Scientific Inc. Waltham, MA) according to the manufacturers’ protocol. Gene expression analysis was conducted at Takara Bio Inc. (Shiga, Japan) using Agilent microarrays (https://catalog.takara-bio.co.jp/jutaku/? _fsi = hl3HYjFg&_fsi = hl3HYjFg&_fsi = hl3HYjFg). The gene-expression profiling was performed on mRNA samples using SurePrint G3 Human GE v3 8 × 60 K Microarray (Agilent Technologies, Santa Clara, USA) to compare the gene signatures between CD34^+^ and CD34^−^ MM cells. GSEA was performed with Ingenuity® Pathway Analysis (IPA) (QIAGEN KK, Tokyo, Japan).

### Network analysis with Ingenuity Pathway Analysis (*IPA*) system

IPA system (Version 39,480,507, Ingenuity Systems; Qiagen, Venlo, Netherlands) uses a network generation algorithm to segment the network map between molecules into multiple networks and assign scores for each network [28, 29]. The score is generated based on hypergeometric distribution, where the negative logarithm of the significance level is obtained by Fisher’s exact test at the right tail. For canonical pathway analysis, the − log (*P*-value) > 2 was taken as threshold, the Z-score > 2 was defined as the threshold of significant activation, while Z-score <  − 2 was defined as the threshold of significant inhibition.

### Statistical analysis

In all experiments, the results were presented as means ± standard error of the mean. Student’s *t* test or the Wilcoxon signed-rank test was used to assess a statistical difference between the two groups and one-way analysis of variance was used to compare three or four groups. Multiple comparisons were analyzed using the Bonferroni correction. *P* values < 0.05 were treated as a statistical significance. A Wilcoxon signed-rank test was used for paired group comparisons. The FlowJo software (version 10.7.1, Ashland, Oregon, USA) was used for flow cytometry analysis. All other statistical analyses were performed using Microsoft Excel software (Microsoft 365, Microsoft, Redmond, WA) and EZR package (version 1.54, Saitama Medical Center, Jichi Medical University, Saitama, Japan).

## Results

**Fig. 1:**
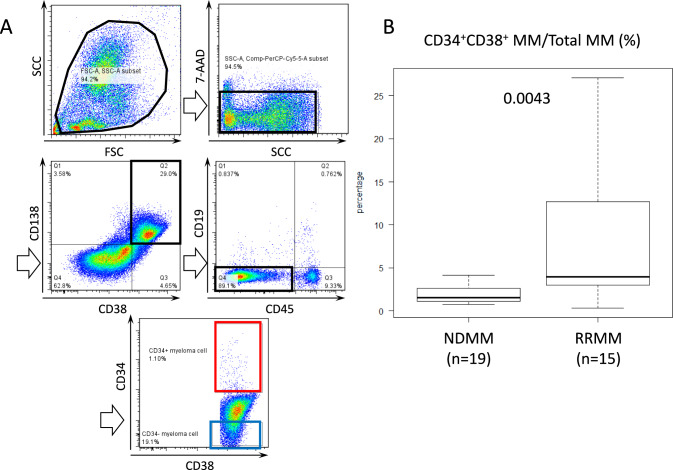
The median percentage of CD34^+^ MM cells was significantly higher in relapse/refractory (RR) than in newly diagnosed (ND) samples. **A** The schema of flow cytometry analysis on the representative patient is shown. Dead cells and doublet samples were excluded. Middle left panel gated CD38^+^CD138^+^ and middle right panel gated CD19^−^CD45^−^ in MM samples was displayed, respectively. Cells gated with CD38^+^CD138^+^CD19^−^CD45^−^ were defined as phenotypic MM cells. **B** When MM cells were divided by CD34, % of CD34.^+^ MM cells in total MM cells was significantly higher in RRMM (*n* = 19) than in NDMM samples (*n* = 15). Comparisons were done using an unpaired, two-tailed student’s *t* test (*P* = 0.0043)

First, we examined the proportion of CD34^+^ MM cells within the total MM cell population (defined as CD45^−^CD19^−^CD38^+^CD138^+^ cells) using 34-MM samples, consisting of 19 NDMM and 15 relapsed/refractory MM (RRMM) cases. As shown in Fig. 1, the median percentage of CD34^+^ MM cells was 1.5% (95% Confidence Interval [CI] 0.72–4.15) in NDMM and 3.98% (95% CI 0.31–27.1) in RRMM, showing a significant increase in the RRMM group.

Next, we performed gene-expression analysis on CD34^+^ and CD34^−^ MM cells MM cells from two NDMM patients (Case 1 and Case 2, as shown in Supplementary Table 1). The gene-expression profiles were analyzed using Gene Set Enrichment Analysis (GSEA) for canonical pathway identification. Through IPA, we identified 14 out of 289 canonical pathways that were significantly upregulated in CD34^+^ MM cells compared to CD34^−^ MM cells, with z-scores greater than 2.0 (Supplementary Table 2), most of which were involved in inflammation and/or immune reactions.

**Fig. 2:**
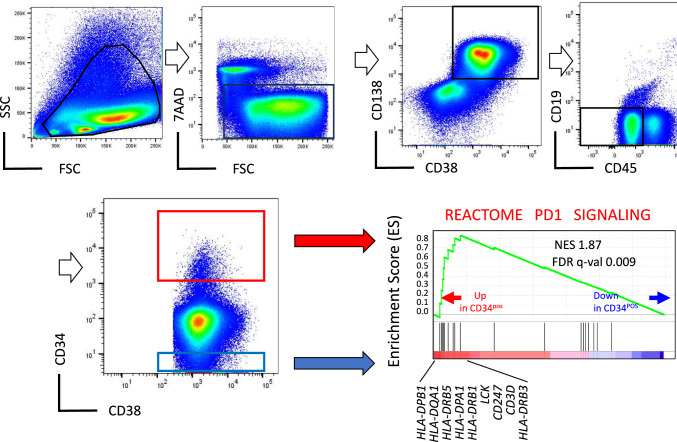
Gene-set enrichment analysis revealed that genes associated with evasion of PD1 signaling are enriched in CD34 + MM cells. Doublet cells were excluded beads on FSC-W/FSC-H and SSC-W/SSC-H values and 7-AAD stain solution was used for discrimination of dead cells from viable cells. After dividing doublets and dead cells, CD38^+^CD138^+^CD19^−^CD45^−^ MM cell fraction was gated and CD34^+^ MM cells were identified. We used isotype controls and fluorescence-minus-one analyses to conduct gating and compensation. In this condition, we utilized 10^3^ fluorescence intensity as the cut-off value of CD34 expression on MM cells (that is, ≥ 10^3^ as CD34^+^ cells and < 10^1^ as CD34^−^ cells). The same method was used in all other experiments. Gene-set enrichment analysis (GSEA) for the PD1sigmaling pathway in CD34^+^ cells and < 10^1^ as CD34^−^ cells. The enrichment score (ES) was calculated according to the original GSEA statistics. Significances are based on the false-discovery rate (FDR < 25%) and indicated by FDR (q value) in the insets of the GSEA plots

We further assessed the expression of genes involved in PD-1 signaling, a critical pathway in anti-tumor immune evasion. The analysis revealed that genes related to PD-1 signaling were significantly upregulated in CD34^+^ MM cells compared to CD34^−^ MM cells, with a normalized enrichment score (NES) of 1.66 (> 0, indicating significance) and a false discovery rate (FDR) *q*-value of 0.009 (*q* < 0.05, significant) (Fig. 2).

**Fig. 3:**
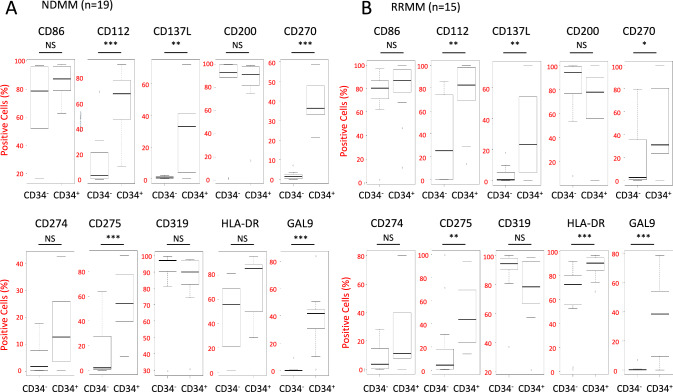
Several immune-checkpoint molecules were more frequently expressed on CD34^+^ MM cells compared to CD34^−^ MM cells. Comparison of the frequency (%) of positive cells of several immune-checkpoint molecules on CD34^+^ MM and CD34.^−^ MM in **A** NDMM (*n* = 12) and **B** RRMM (*n* = 15). The immune-checkpoint molecules examined were CD112, CD86, CD275, GAL9, CD270, CD274, HLA-DR, CD319, CD137L, and CD200. Plots (generated using EZR package (version 1.54)) show the range of data values obtained. Top and bottom whiskers, values of the top and bottom 25% of the cases, respectively; boxed area, interquartile range and the significant P values between groups; horizontal black line, median value e; circles, outlying values (as defined by EZR). Comparisons were done using an unpaired, two-tailed student’s *t* test (*P < .05, **P < .01, ***P < .001, *NS* Not significant)

We next compared the expression rates (%) of 10 immune-checkpoint molecules (CD86, CD112, CD137 ligand [L], CD200, CD270, CD274, CD275, CD319, HLA-DR, and GAL9) between CD34^+^ and CD34^−^ MM cells from 19 NDMM patients using flow cytometry. As shown in Fig. 3A, CD112, CD137L, CD275, CD270, and GAL9 were expressed at significantly higher rates in the CD34^+^ MM cell fraction compared to the CD34^−^ MM cell fraction (*P* < 0.01). No significant differences were observed in the expression rates of the other molecules, and none of the molecules were expressed at higher rates in the CD34^−^ MM cells than in the CD34^+^ MM cells. We then performed the same analysis on 15 RRMM patients. As shown in Fig. 3B, CD34^+^ MM cells expressed CD112, CD137L, CD270, CD275, HLA-DR, and GAL9 at significantly higher rates than CD34^−^ MM cells (*P* < 0.01).

To investigate the clinical significance of these molecules, we compared their expression rates between NDMM and RRMM in the CD34^+^ MM cell fraction. However, none of the molecules showed a significant difference in their expression rates between NDMM and RRMM (Supplementary Fig. 1A).

We further analyzed the relationship between their expression rates and clinical statuses, including newly diagnosed (ND, *n* = 12), resistant to lenalidomide (R, *n* = 3), resistant to bortezomib (Bor, *n* = 3), and resistant to both lenalidomide and bortezomib (RBor, *n* = 3), in the CD34^+^ MM population. The mean expression rates of these molecules did not correlate with resistance to Len and/or Bor due to significant patient-to-patient variations (Supplementary Fig. 1B).

**Fig. 4:**
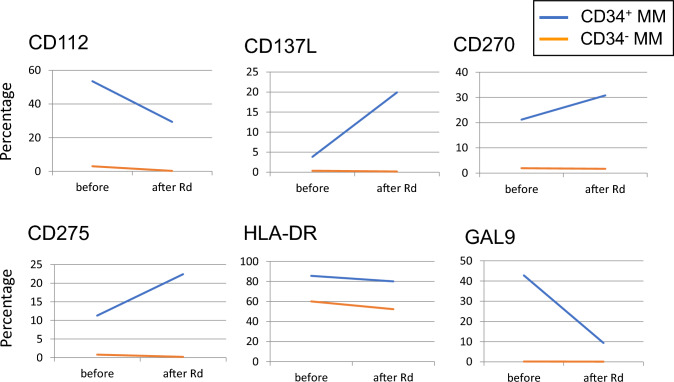
Immune phenotype on CD34^+^ MM cells would be altered by treatment. The expression rates of CD112, CD137L, CD270, CD275, HLA-DR, and GAL9 on CD34^+^ and CD34^−^ MM cells in a representative patient (case 12) before and after the treatment with lenalidomide was shown

To gain further insights, we analyzed the expression rates before and after Rd (lenalidomide and dexamethasone) treatment in a single patient (Case 9). The frequency of HLA-DR^+^ cells was not affected by Rd treatment in either CD34^+^ or CD34^−^ MM cells (Fig. 4). While the expression of CD112, CD137L, CD270, CD275, and GAL9 was barely detectable ion CD34^−^ MM cells both before and after Rd treatment, these molecules were expressed in a significant proportion of CD34^+^ MM cells before treatment. Notably, the fractions positive for CD137L, CD270, and CD275 increased, while those positive for CD112 and GAL9 decreased in the CD34^+^ MM cell population after Rd treatment. These results suggest a substantial difference in the immune phenotype between CD34^+^ and CD34^−^ MM cells and indicate that the immune phenotype of CD34^+^ MM cells may be altered by MM treatment.

Based on the finding that CD112, CD137L, CD270, CD275, HLA-DR, and GAL9 were more frequently expressed on CD34^+^ MM cells compared to CD34^−^ MM cells in NDMM and/or RRMM, we next analyzed the expression of their corresponding partner molecules on BM CD3^+^ T cells in 5 NDMM patients and 5 healthy donors (HD) using flow cytometry. The molecules analyzed on T cells were as follows: TIGIT as the partner for CD112, CD137 for CD137L, BTLA for CD270, CD278 for CD275, LAG3 for HLA-DR, and Tim-3 for GAL9 [11].

**Fig. 5:**
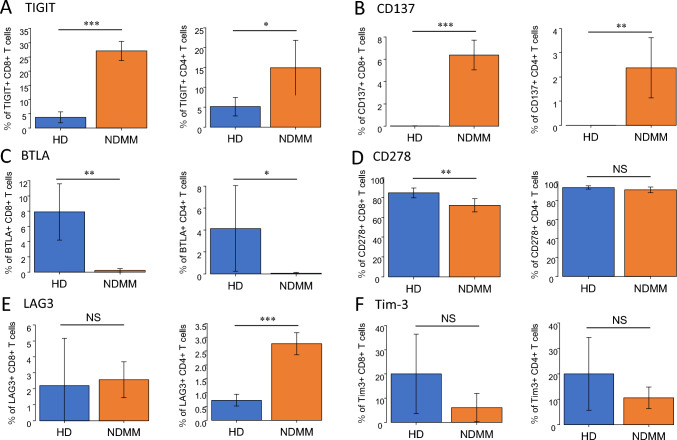
CD8.^+^ T-cell subsets in the bone marrow of NDMM showed high expression of TIGIT and CD137**.** CD3 T cells were isolated using magnetic separators for cell separation, and immune-checkpoint molecules on T cells subsets were analyzed by Flow cytometry. The immune-checkpoint molecules on the T cell side correspond to TIGIT with CD112 (**A**), CD137 with CD137L (**B**), BTLA with CD270 (**C**), CD278 with CD275 (**D**), LAG3 with HLA-DR (**E**), and Tim-3 with GAL9 (**F**) in CD4 and CD8 T cells, respectively. We examined the expression rates of their immune checkpoints on the surface of T cells in the bone marrow of 5 NDMM patients and 5 healthy donors as controls. Data are presented as mean ± SEM. Comparisons were done using an unpaired, two-tailed student’s *t* test (*P < .05, **P < .01, ***P < .001, *NS* Not significant)

Both CD4^+^ and CD8^+^ T cells from NDMM patients more frequently expressed TIGIT and CD137 compared to those from HD (*P* < 0.001, Fig. 5A, B). In addition, CD4^+^ T cells but not CD8^+^ T from NDMM expressed LAG3 more frequently than those from HD (*P* < 0.001 and not significant [NS], respectively, Fig. 5E). In contrast, both CD4^+^ and CD8^+^ T cells from HD more frequently expressed BTLA than those from NDMM (*P* < 0.001, Fig. 5C). No significant differences were observed in the expression rates of CD278 or Tim-3 between NDMM and HD in either CD4^+^ or CD8^+^ T cells (Fig. 5D, F). These results suggest that the anti-MM activity of CD3^+^ T cells against CD34^+^ MM cells may be suppressed through CD112/TIGIT and CD137L/CD137 interactions.

**Fig. 6:**
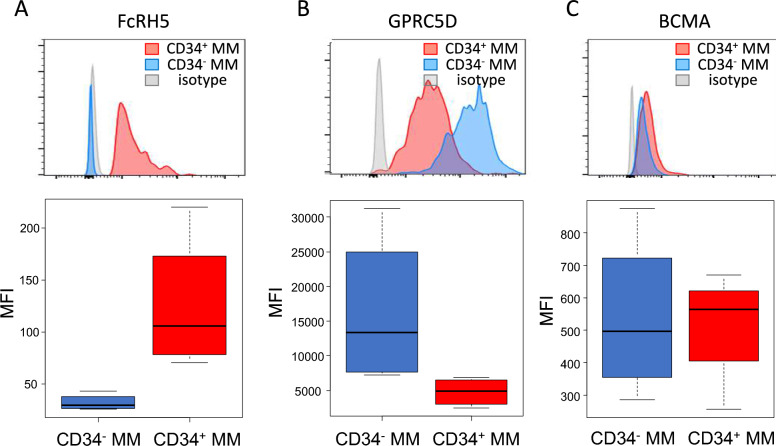
Differential expression of antigens targeted by bispecific Abs in multiple myeloma on CD34^+^ MM cells**.** The expression of the antigens targeted by the bispecific Abs against MM was compared between CD34^+^ MM cells and CD34^−^ MM cells. The results from the representative patient are shown (**A–C**, upper, Red-filled line and blue-filled line, expression of each molecule on CD34^+^ MM cells and CD34^−^ MM cells, respectively; gray-filled areas, isotype control Ab staining on whole MM cells). The Mean fluorescence intensities (MFIs) of bispecific Ab-related markers (BCMA, GPRC5D, FcRH5) in CD34^+^ MM cells and CD34.^−^ MM cells are indicated (*n* = 4). (**A–C**, lower) The MFIs of representative patients are shown in a histogram. Data are presented as mean ± SEM. Comparisons were done using an unpaired, two-tailed student’s *t* test (*P < .05, *NS* Not significant)

To gain insight into the efficacies of currently utilized CAR-T therapies and bispecific Abs, both of which engage T cells and MM cells through the binding of CD3 on T cells to the specific antigens on MM cells, such as BCMA [30, 31], GPRC5D [32, 33], and FcRH5 (also known as FcRL5) [34, 35], we examined their expression on CD34^+^ and CD34^−^ MM cells from 4 NDMM patients. As shown in Fig. 6A, FcRH5 expression levels (MFI) were significantly higher on CD34^+^ MM cells compared to CD34^−^ MM cells (*P* < 0.05), while GPRC5D was more intensely expressed on CD34^−^ MM cells than CD34^+^ MM cells (p < 0.05) (Fig. 6B). There was no significant difference in BCMA expression between CD34^+^ and CD34^−^ MM cells (Fig. 6C). Based solely on their expression levels, it can be considered that Abs targeting FcRH5 might be more effective against CD34^+^ MM cells compared to CD34^−^ MM cells, while those targeting GPRC5D might be less effective for this cell population. However, this hypothesis needs to be confirmed through clinical trials.

## Discussion

We previously reported CD34^+^ MM cells constitute only 2.20% of total MM cells in NDMM patients, but this fraction increased to 42.6% of MRD samples from MM patients, who achieved good responses (equal to or deeper than very good partial remission) [21]. Also, CD34^+^ MM cells accounted for 17.7% of the RRMM cell population. However, in the current study, CD34^+^ MM cells constituted only 3.98% of the RRMM population (Fig. 1), which seems inconsistent with our previous findings. Moreover, in this study, the patient-to-patient variation was substantial, ranging from 0.32% to 27.1%. We hypothesize that this discrepancy, along with the large variation, may stem from differences in the timing of sample collection from RRMM patients. CD34^+^ MM cells are more immature than CD34^−^ MM cells and are capable of generating CD34^−^ MM cells in xenotransplantation models [21, 22]. Thus, we believe that CD34^+^ MM cells represent a more critical target population than CD34^−^ MM cells and should be eliminated to improve the prognosis of MM and potentially achieve a cure.

Recent studies have elucidated that increased expression of PD-L1 on MM cells, as well as PD-1 and CTLA-4 on T cells, is associated with unfavorable outcomes in MM patients [17]. These findings have sparked interest in investigating the potential of ICIs as a novel therapeutic approach against MM. However, despite promising preclinical rationales, several clinical studies have yielded negative results. In the KEYNOTE 013 phase Ib trial, pembrolizumab monotherapy failed to induce a response in any of the 30 patients with relapsed/refractory MM (RRMM), with a median progression-free survival (PFS) of only 2.7 months [36]. Similarly, in a phase I study of nivolumab monotherapy for RRMM, no patients achieved a response, and the median PFS was 2.5 months [37].

Preclinical studies have shown that ICIs combined with IMiDs demonstrate synergistic effects, prompting numerous trials evaluating the efficacy and safety of the ICI and IMiD combination [38]. However, these combinations did not lead to improvements in clinical outcomes for MM patients, including overall response rates, progression-free survival, and overall survival. Furthermore, the increased frequency of serious adverse events has been a significant concern [39–42]. To utilize ICIs more effectively, we analyzed the immune profile of CD34^+^ and CD34^−^ MM cells. Our results revealed that CD34^+^ MM cells exhibited high expression of CD112, CD137L, CD270, CD275, HLA-DR, and GAL9. In addition, CD4^+^ and CD8^+^ T cells from NDMM patients more frequently expressed TIGIT and CD137.

TIGIT (T-cell immunoglobulin and ITIM domains) is expressed on T cells and NK cells and acts as an inhibitory receptor by binding to its ligands: CD112 (also known as PVRL2 or NECTIN2), CD155 (PVR), and CD113 (PVRL3 or NECTIN3) [43]. Among these, CD155 has a higher affinity for TIGIT compared to CD112, making it the primary ligand for TIGIT. TIGIT competes with CD226 (DNAM1) for binding to both CD155 and CD112 [44]. By engaging these ligands, TIGIT inhibits T-cell activity and is involved in or related to T-cell exhaustion [45]. In addition, TIGIT enhances the function of Tregs [46, 47], indicating its immunosuppressive role in cancer biology.

In this study, we found that TIGIT was expressed at higher levels on both CD4^+^ and CD8^+^ T cells from MM patients compared to those from HD. Our results align with previous findings from the previous mouse model, where the frequency of TIGIT^+^ CD8^+^ T cells was positively correlated with tumor burden, while the frequency of CD226^+^ CD8^+^ T cells showed a negative correlation [48]. In this mouse model, both anti-TIGIT and anti-PD-1 antibodies significantly prolonged remission after stem cell transplantation. In addition, in another study, anti-TIGIT antibody treatment significantly reduced tumor volume by restoring CD8^+^ T-cell function, thereby improving survival in treated mice [49]. However, it is important to note that in this model, anti-PD-1 Ab treatment did not show a survival benefit compared to controls.

Based on these preclinical data, anti-TIGIT mAbs are being investigated in several phase 1/2 clinical trials, either as monotherapy or in combination with anti-PD-1/PD-L1 Abs or chemotherapy, for the treatment of malignant lymphoma and MM [50, 51]. In the present study, we found that PD-1 pathways were more active in CD34^+^ MM cells compared to CD34^−^ MM cells, while CD112 was expressed at similar levels on both CD34^+^ and CD34^−^ MM cells. Thus, further analysis is required to evaluate the efficacy of anti-TIGIT mAbs, with or without PD-1/PD-L1 blockade, on CD34^+^ and CD34^−^ MM cells to clarify the significance of the enhanced PD-1 pathways in CD34^+^ MM cells.

It has been reported that there is a difference in CD137L expression between MGUS and MM, with significantly increased expression in the MM group [52]. However, we found that CD137L expression was upregulated on CD34^+^ MM cells, but hardly detectable on CD34^−^ MM cells, indicating the heterogeneity of MM cells in terms of CD137L expression. Furthermore, it has been reported that CD137L expression decreased in patients who achieved good responses (partial response [PR] or better) after treatment [52], suggesting that CD137L may be involved in the development of MM and could contribute to therapy resistance.

On the other hand, CD137 is expressed on various immune cells, including T and NK cells, and acts as a potent costimulatory molecule of the tumor necrosis factor receptor (TNFR) superfamily, which is expressed on activated cells [53, 54]. In addition, we found that CD137 was detected on both CD4^+^ and CD8^+^ T cells from NDMM patients, while it was scarcely detected on those from HD. CD137 signaling in T cells promotes their clonal expansion, differentiation, and survival, thereby enhancing the anti-cancer activity of T cells. Moreover, a CD137 agonist has been reported to augment the anti-PD-1 Ab-mediated restoration of exhausted CD8^+^ T cells [55]. Based on these results, two phase 1 clinical trials using mAbs against CD137 (urelumab and utomilumab) were conducted in advanced cancer patients (including those with B-cell non-Hodgkin lymphoma). However, these trials were halted due to severe hepatotoxicity in the case of urelumab and low efficacy in the case of utomilumab [56]. Regarding MM, anti-CD137 agonistic mAb treatment significantly reduced systemic tumor burden in a murine syngeneic disseminated myeloma model (5TGM1), which closely resembles human MM [57, 58], suggesting the potential utility of anti-CD137 agonistic mAbs. Further comprehensive studies are necessary to elucidate the roles of the enhanced expression of CD137L on CD34^+^ MM cells and CD137 on T cells in the context of anti-MM immune responses. Also, further efforts are required to improve the efficacy and ensure the safety of anti-CD137 agonistic mAbs.

In this study, we clarified the immune profiles of both CD34^+^ and CD34^−^ MM cells. These findings provide valuable insights for selecting optimal immunotherapy targets in MM. Our results also suggest that ICIs targeting CD112/TIGIT and CD137L/CD137 interactions might be promising in MM treatment. However, more comprehensive studies and clinical trials are essential to validate our hypothesis.

## Supplementary Information

Below is the link to the electronic supplementary material.

Supplementary file1 (PPTX 262 KB)

Supplementary file2 (XLSX 14 KB)

Supplementary file3 (XLSX 12 KB)

Supplementary file4 (DOCX 19 KB)
