# Supplementary material for: CD34+ and CD34− MM cells show different immune-checkpoint molecule expression profiles: high expression of CD112 and CD137 ligand on CD34+ MM cells
Source: Int J Hematol. 2024 Nov 12;121(1):89–99. doi: 10.1007/s12185-024-03867-0 (PMC11742359; doi:10.1007/s12185-024-03867-0)
Supplement: Supplementary file 1 — Supplementary file1 (PPTX 262 KB) [file 12185_2024_3867_MOESM1_ESM.pptx]

## Slide 1
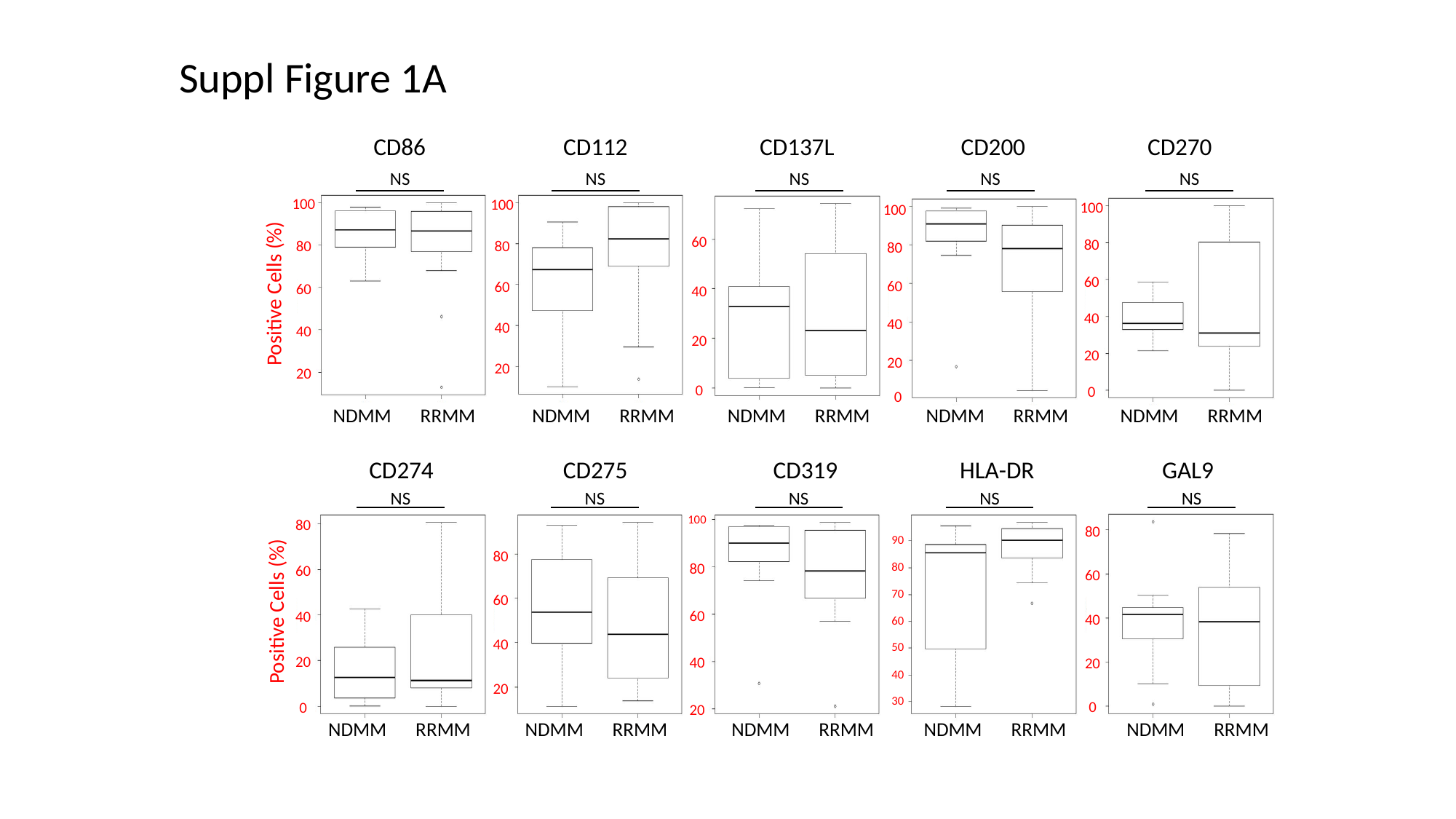

Suppl Figure 1A
CD86
CD112
CD137L
CD200
CD270
NS
NS
NS
NS
NS
100
100
100
100
60
80
80
80
80
60
60
60
60
Positive Cells (%)
40
40
40
40
40
20
20
20
20
20
0
0
0
NDMM　RRMM
NDMM　RRMM
NDMM　RRMM
NDMM　RRMM
NDMM　RRMM
CD274
CD275
CD319
HLA-DR
GAL9
NS
NS
NS
NS
NS
100
80
80
90
80
80
80
60
60
70
60
Positive Cells (%)
60
40
40
60
40
50
20
40
20
40
20
30
0
0
20
NDMM　RRMM
NDMM　RRMM
NDMM　RRMM
NDMM　RRMM
NDMM　RRMM

## Slide 2
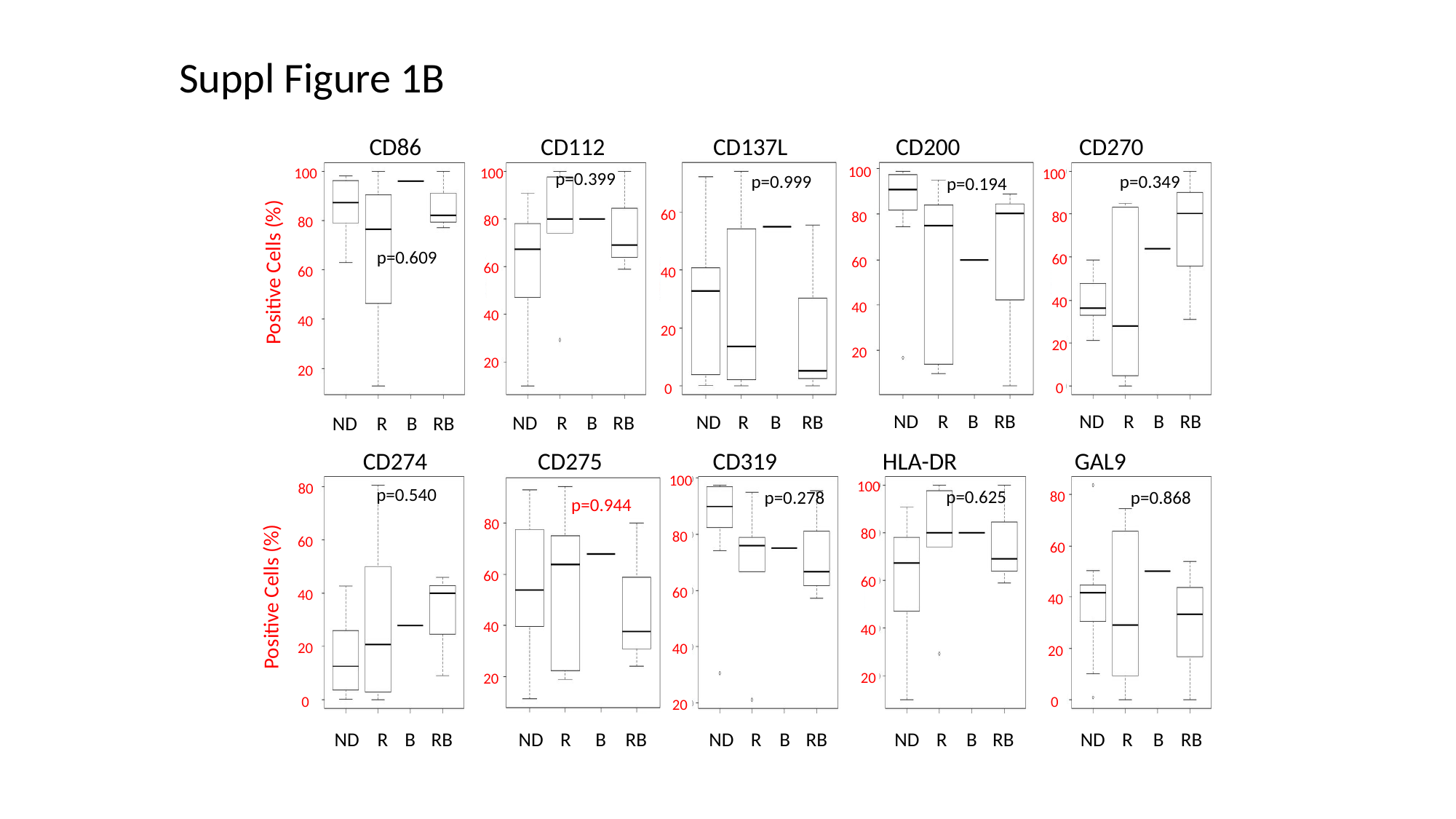

Suppl Figure 1B
CD86
CD112
CD137L
CD200
CD270
CD274
CD275
CD319
HLA-DR
GAL9
100
100
100
100
p=0.399
p=0.999
p=0.349
p=0.194
60
80
80
80
80
p=0.609
60
60
60
Positive Cells (%)
60
40
40
40
40
40
20
20
20
20
20
0
0
RB
ND
R
B
RB
ND
R
B
RB
ND
R
B
RB
ND
R
B
RB
ND
R
B
100
100
80
p=0.540
p=0.625
p=0.868
p=0.278
80
p=0.944
80
80
80
60
60
60
60
60
Positive Cells (%)
40
40
40
40
20
40
20
20
20
0
0
20
RB
ND
R
B
RB
ND
R
B
RB
ND
R
B
RB
ND
R
B
RB
ND
R
B
