# Supplementary material for: CD34+ and CD34− MM cells show different immune-checkpoint molecule expression profiles: high expression of CD112 and CD137 ligand on CD34+ MM cells
Source: Int J Hematol. 2024 Nov 12;121(1):89–99. doi: 10.1007/s12185-024-03867-0 (PMC11742359; doi:10.1007/s12185-024-03867-0)
Supplement: Supplementary file 4 — Supplementary file4 (DOCX 19 KB) [file 12185_2024_3867_MOESM4_ESM.docx]

**Supplemental Methods**

The cells were stained with the following Ab combinations of human antigens for 30 minutes at 4℃: FITC-conjugated anti-CD38 Ab (LD38, Cytognos, Salamanca, Spain), BV510-conjugated anti-CD138Ab (MI15, BioLegend, San Diego, USA), PE-Cy7-conjugated anti-CD19 Ab (J3-119, Beckman coulter, Marseille, France), Pacific Blue-conjugated anti-CD45 Ab (T29/33, Dako, Glostrup, Denmark), Alexa Fluor700-conjugated anti-CD34 Ab (581, BioLegend), PE-conjugated anti-CD112 Ab (TX31, BioLegend), PE-CF594-conjugated anti-CD86 Ab (2331, BD Biosciences), BV711-conjugated anti-CD275 Ab (2D3/B7-H2, BD Biosciences), PE-conjugated anti-Galectin-9 Ab (9M1-3, BioLegend), BV605-conjugated anti-HVEM (CD270) Ab (CW10, BD Biosciences), BV786-conjugated anti-CD274 Ab (M1H1, BD Biosciences), BV786-conjugated anti-HLA-DR Ab (G46-6, BD Biosciences), PerCP/Cy5.5-conjugated anti-CD319 Ab (162.1, BioLegend), BV605-conjugated anti-CD137L Ab (C65-485, BD Biosciences), BV711-conjugated anti-CD200 Ab (MRC OX-104, BD Biosciences), APC-conjugated anti-CD4 Ab (SK3, BioLegend), Alexa Fluor700-conjugated anti-CD8 Ab (SK1, BioLegend), PE-conjugated anti-CD137 Ab (4B4-1, BD Biosciences), APC/Cy7-conjugated anti-CD278 (ICOS) Ab (C398.4A, BioLegend), BV421-conjugated anti-CD272 (BTLA) Ab (J168-540, BD Biosciences), PE-conjugated anti-LAG3 (CD223) Ab (T47-530, BD Biosciences), APC/Cy7-conjugated anti-CD366 (Tim3) Ab (F38-2E2, BioLegend), and BV510-conjugated anti-TIGIT Ab (741182, BD Biosciences), PE-conjugated anti-BCMA (CD269) Ab (19F2, BioLegend), APC-conjugated anti-FcRL5 (CD307e) Ab (509f6, BioLegend), and APC-conjugated anti-GPRC5D Ab (19, Sino Biological, Beijing, China) were used according to the manufactures’ instructions. PE-conjugated Mouse IgG2a kappa (MOPC-173, BD Biosciences), APC-conjugated Mouse IgG2a kappa (G155-178, BD Biosciences), and APC-conjugated Mouse IgG2b kappa (27-35, BD Biosciences) were used as isotype control.

**Supplemental figure 1.**

**There was no correlation between the disease status and changes in expression of immune checkpoint molecules on CD34+ MM cells.**

(A) The expression ratio (%) of various immune checkpoint molecules on CD34+ MM cells between NDMM (n=12) and RRMM (n=15) were compared.

(B) Comparison of the percentage of positive cells of immune checkpoint-related molecules on CD34+ MM cells at ND (n=12), post-Len treatment resistance (R, n=3), post-Bor treatment resistance (B, n=3), and post-Len+Bor treatment resistance (RB, n=3) are shown. Plots (generated using EZR package (version 1.54)) show the range of data values obtained. Top and bottom whiskers, values of the top and bottom 25% of the cases, respectively; boxed area, interquartile range and the significant P values between groups; horizontal black line, median value; circles, outlying values (as defined by EZR). Comparisons were done using an unpaired, two-tailed student’s t test (*P<.05, **P<.01, ***P<.001, NS: not significant).

**Supplemental table 1.** Characteristics of the patients analyzed with flow cytometry for phenotypic multiple myeloma cells in all experiments

Abbreviations. NDMM: newly diagnosis multiple myeloma. RRMM: relapse and refractory multiple myeloma. Vd: bortezomib, dexamethasone. ASCT: autologous stem cell transplantation. Rd: lenalidomide, dexamethasone. VCD: bortezomib, cyclophosphamide, dexamethasone. IRd: ixazomib, lenalidomide, dexamethasone. IXA: ixazomib. Pd: pomalidomide, dexamethasone. Kd: carfilzomib. neg: negative, NA: not assesment.

**Supplemental table 2.** Gene set enrichment analysis for the canonical pathway using the ingenuity pathways analysis system
